# Combination Therapy Accelerates Diabetic Wound Closure

**DOI:** 10.1371/journal.pone.0092667

**Published:** 2014-03-20

**Authors:** Robert J. Allen Jr., Marc A. Soares, Ilyse D. Haberman, Caroline Szpalski, Jeffrey Schachar, Clarence D. Lin, Phuong D. Nguyen, Pierre B. Saadeh, Stephen M. Warren

**Affiliations:** Institute of Reconstructive Plastic Surgery, New York University Langone Medical Center, New York, New York, United States of America; Cedars-Sinai Medical Center; UCLA School of Medicine, United States of America

## Abstract

**Background:**

Non-healing foot ulcers are the most common cause of non-traumatic amputation and hospitalization amongst diabetics in the developed world. Impaired wound neovascularization perpetuates a cycle of dysfunctional tissue repair and regeneration. Evidence implicates defective mobilization of marrow-derived progenitor cells (PCs) as a fundamental cause of impaired diabetic neovascularization. Currently, there are no FDA-approved therapies to address this defect. Here we report an endogenous PC strategy to improve diabetic wound neovascularization and closure through a combination therapy of AMD3100, which mobilizes marrow-derived PCs by competitively binding to the cell surface CXCR4 receptor, and PDGF-BB, which is a protein known to enhance cell growth, progenitor cell migration and angiogenesis.

**Methods and Results:**

Wounded mice were assigned to 1 of 5 experimental arms (n = 8/arm): saline treated wild-type, saline treated diabetic, AMD3100 treated diabetic, PDGF-BB treated diabetic, and AMD3100/PDGF-BB treated diabetic. Circulating PC number and wound vascularity were analyzed for each group (n = 8/group). Cellular function was assessed in the presence of AMD3100. Using a validated preclinical model of type II diabetic wound healing, we show that AMD3100 therapy (10 mg/kg; i.p. daily) alone can rescue diabetes-specific defects in PC mobilization, but cannot restore normal wound neovascularization. Through further investigation, we demonstrate an acquired trafficking-defect within AMD3100-treated diabetic PCs that can be rescued by PDGF-BB (2 μg; topical) supplementation within the wound environment. Finally, we determine that combination therapy restores diabetic wound neovascularization and accelerates time to wound closure by 40%.

**Conclusions:**

Combination AMD3100 and PDGF-BB therapy synergistically improves BM PC mobilization and trafficking, resulting in significantly improved diabetic wound closure and neovascularization. The success of this endogenous, cell-based strategy to improve diabetic wound healing using FDA-approved therapies is inherently translatable.

## Introduction

Diabetic foot ulceration not only affects an individual's physical functioning, psychosocial wellbeing, and quality of life, but it also financially impacts the US healthcare system [1]. According to data from the Centers for Disease Control, diabetics have an estimated 25% lifetime risk of developing a foot ulcer; and, compared to euglycemic patients, they have more than a 100 times greater risk of suffering a lower extremity amputation [2]. Each year, nearly 83,000 lower extremity amputations are performed for nonhealing diabetic foot ulcers. Alarmingly, diabetic ulcer-related amputations not only result in limb loss, but they also contribute to a 3-year mortality rate of 75.9% [3].

Since the worldwide prevalence of diabetes is expected to grow to 4.4% (438 million) by 2030, the burden of diabetic wounds can be expected to increase accordingly [2], [4], [5]. Current diabetic wound treatment hinges on patient education, prevention, and early diagnosis. However, once a wound has developed, invasive therapies are costly while noninvasive therapies are less effective [6]. Ultimately, since current treatments do not correct the underlying pathophysiology, many patients suffer untoward complications and require amputations [4].

Although the pathogenesis of diabetic wound healing is multifactorial, impaired neovascularization is a central element [7]. Recent evidence demonstrates that bone marrow (BM)-derived progenitor cells (PCs) play an integral role in new blood vessel formation at sites of injury [8], [9]. Specifically, cutaneous injury stimulates BM PC mobilization. Circulating PCs (cPCs) then traffic to injury sites, transmigrate into the tissues, and contribute to new vessel formation [8], [10]. Recently, we have demonstrated that while there is no difference in the number of BM PCs in diabetic and wild-type mice, there are fewer cPCs in diabetic mice at baseline and in response to peripheral injury [11]. Based on this finding, we hypothesized that impaired diabetic wound healing may be partially attributed to decreased PC mobilization, migration/homing, and/or function [12], [13]. We tested this hypothesis by harvesting BM PCs and adoptively transferring them topically or subcutaneously into diabetic wounds [14]. This and other studies demonstrated that adoptive cellular therapy can overcome impaired PC mobilization, homing, and migration and significantly improves diabetic wound healing [14], [15]. While adoptive PC treatment of diabetic wounds is effective, the translation of this technology to clinical practice is fraught with challenges [16] For this reason, we currently are focused on strategies to enhance endogenous mechanisms of PC mobilization and trafficking. Using two FDA-approved compounds, we hypothesize that endogenously mobilizing BM PCs into the circulation with the CXCR4 antagonist, AMD3100, and improving PC homing to the wound with topical platelet-derived growth factor-BB (PDGF-BB) will improve diabetic wound closure.

## Methods

### Ethics Statement

The use of animals in this study was approved by the NYU Langone Medical Center Animal Care & Use Program under IACUC protocol #061104. Furthermore, all experiments were performed in accordance with the guidelines set forth by the NYU Langone Medical Center Animal Care & Use Program.

### Mice and Wounding Model

C57BL/6J (#664) wild-type (wt, n = 8) and type II diabetic mice (n = 32) homozygous for Leprdb/db (#642) aged 8–12 weeks were purchased from Jackson Laboratories (Bar Harbor, ME). Blood glucose was assessed using an AccuCheck Advantage glucometer and AccuCheck Comfort Strips (Roche; Branchburg, NJ). Mice were anesthetized by intramuscularly administering 0.5–0.7 mL/kg of “Rodent Anesthesia Cocktail” that consisted of ketamine (50–70 mg/kg), xylazine (7.5–10.5 mg/kg) and acepromazine (4.15–5.81 mg/mL). Once adequate anesthesia was obtained, two full-thickness wounds (6.2±0.1 mm) were created on the dorsum of mice using a sterile 6-mm punch biopsy as previously described [17]. A silicone stent (Johnson & Johnson; New Brunswick, NJ) was secured to the wound perimeter to prevent contraction and allow healing by secondary intention. The stented wound was tattooed with India Ink and covered with a clear occlusive dressing (3 M; St. Paul, MN). Standardized photographs were taken every 7 days. Wound area was measured digitally (Photoshop CS3, Adobe Systems, Inc.; San Jose, CA) and calibrated against the internal diameter of the silicon stent to correct for magnification, perspective, or parallax effects. Time to wound closure (number of days for complete re-epithelialization) and percent wound closure (1-([wound area]/[original wound area])) were measured photogrammetrically.

At the conclusion of the study period or prior to the harvesting of mouse wounds, peripheral blood or bone marrow, all animals were euthanized by CO2 narcosis.

### Treatment Groups

Mice were assigned to one of 5 experimental groups (n = 8/group): saline treated wild-type mice, saline treated diabetic mice, PDGF-BB treated diabetic mice, AMD3100 treated diabetic mice, and AMD3100/PDGF-BB treated mice. Daily treatment with AMD3100 (10 mg/kg, i.p.; Genzyme Corp., Cambridge, MA) and/or PDGF-BB (2 μg/wound, topical; 0.01% gel; Johnson & Johnson; New Brunswick, NJ) began on post-wounding day 3 and continued until wound closure.

### Isolation of Mononuclear Cells (MNCs) from Peripheral Blood and Bone Marrow

Peripheral blood (PB) was harvested from mice (n = 8 per group) at baseline, 7, 14, and 21 days post-wounding 1-hour following treatment with AMD3100 or sterile saline. Bone marrow was flushed from mouse long bones using PBS/10%FBS/5% EDTA as previously described [9], [18]. Mononuclear cells (MNCs) from the peripheral blood and BM were isolated by density gradient centrifugation using Histopaque 1083 (Sigma-Aldrich; St. Louis, MO).

### Flow Cytometry and Isolation of Progenitor Cells

PCs were isolated from bone marrow and peripheral blood MNCs by magnetic cell separation using a commercially available mouse lineage depletion kit (Miltenyi Biotec, Inc.; Auburn, CA). Using this kit, lineage positive cells are removed, leaving an enriched, heterogenous lineage negative (lin-) cell population.

For characterization by flow cytometry, lin- cells were labeled with rat anti-mouse antibodies (fluorescein isothiocyanate-conjugated Sca-1, allophycocyanin-conjugated c-kit, strepavidin-PE-conjugated-Cy7)(BD Bioscience; San Jose, CA and Miltenyi Biotech). All antibodies were titrated and optimized for appropriate detection. Samples were collected using a BD FACSCaliber flow cytometer (Becton-Dickinson; Franklin Lakes, NJ), and analyses were performed with FlowJo 8.0 software (TreeStar Inc.; Ashland, OR).

### Cell Culture

Primary diabetic fibroblasts from dorsal skin were expanded in standard culture media (DMEM/10%FBS/1%antibiotic-antimycotic) (BD Biosciences; San Jose, CA) as described previously [17], [19]. Passages 2–4 were used for all assays.

Isolated lin- cells were stained with FITC-Sca-1, APC-c-kit and sorted using a Dako MoFlo cell sorter (Dako Colorado Inc.; Fort Collins, CO). Enriched lin-/Sca-1+/c-kit+ cells (L-S+K+) were seeded onto 24-well plates (1,000 cells/well) (Corning Costar, Lowell, MA) and expanded in StemSpan Serum-Free media (Stem Cell Technologies; Vancouver, BC, Canada) supplemented with thrombopoietin [TPO: 20 ng/mL], stem cell factor [SCF: 100 ng/mL], interleukin-6 [IL-6: 20 ng/mL], vascular endothelial growth factor [VEGF: 50 ng/mL], and Flt-3 [100 ng/mL] (Peprotech; Rocky Hill, NJ). The L-S+K+ cell population is heterogenous, but enriched for vasculogenic PCs [11]. Supplemented StemSpan was considered vasculogenic PC growth medium. All assays were performed on primary cultured PCs following 7 days of expansion.

### Chemotaxis Assay

PC and fibroblast migration was measured using a modified Boyden chamber assay as previously described [20]. Briefly, SDF-1α (100 ng/mL), PDGF-BB (100 ng/mL) or FBS (control) in vasculogenic PC growth medium or standard cell growth media was placed in the bottom of a 24-well plate. Cells (5×104) ± AMD3100 (5–50 ng/mL) were seeded onto fibronectin-coated (5 μg/cm2) transwell inserts. After 20 hours cells were harvested from the bottom chambers, washed, and centrifuged. Cell pellets were frozen at −80C. Frozen cells were re-suspended in CyQuant Green Fluorescent dye (Invitrogen) and the relative fluorescence was measured using a Synergy TM HT microplate reader (BioTek; Winooski, VT).

### Adhesion Assay

Adhesion of diabetic PCs and fibroblasts was measured in AMD3100 (50 ng/mL) ± PDGF-BB (100 ng/mL). PCs and fibroblasts (1×105 cells/chamber) were added to 4 well chamber slides (Fisher Scientific; Pittsburgh, PA) coated with fibronectin (5 μg/cm2) (Sigma) and incubated at 37°C for 2 hours. Following incubation, non-adherent cells were removed before adherent cells were fixed with 1% paraformaldehyde. Adherent cells were stained with DAPI (4′,6-diamidino-2-phenylindole) (VectaShield; Vector Laboratories, Burlingame, CA) and viewed on an Olympus BX51 epifluorescent microscope. Adobe Photoshop CS3 (Adobe Systems; San Jose, CA) was used to quantify the number of cells/random high-powered field (hpf) under 100× magnification. A total of 6 hpf/group was analyzed for comparison between experimental groups.

### Proliferation Assay

Proliferation of PCs or fibroblasts was measured using BrdU (5-Bromo-2′ deoxy-uridine) labeling and fluorescent detection (Synergy TM HT microplate reader: BioTek; Winooski, VT). Proliferation was compared in media containing AMD3100 (50 ng/ml), PDGF-BB (100 ng/ml), SDF-1α (100 ng/ml), or a combination of the three.

### Histology and Immunofluorescence

Wounds were harvested on day 28 for analysis. Frozen sections were stained with rat anti-mouse CD31 (PECAM; BD Biosciences) primary antibody and goat anti-rat IgG secondary (Alexafluor 594; Invitrogen). Control samples were prepared without primary antibody. Slides were mounted with DAPI (Sigma) and viewed on an Olympus BX51 epifluorescent microscope. DAPI was used to determine the sample outline; whereas, immunofluorescent CD31 staining identified vascular structures (red staining) within the sample. Dual filter images were superimposed to illustrate wound architecture and vascular staining. Adobe Photoshop CS3 was used to segment and quantify positive CD31 staining. Superimposed images revealed vascular staining (CD31 red staining) and cellular nuclei (DAPI blue staining). Vessel density was determined as the molecules of equivalent soluble fluorochrome (MESF) per low power field (LPF) averaged across five consecutive fields using the Nikon NIS Elements software (Nikon, Melville, NY) [21]. All experiments were performed in triplicate by a blinded reviewer.

### Wound Vasculogenesis Assay

To demonstrate the role of PC mobilization and trafficking in neovascularization, 2.5×104 BM-derived diabetic PCs were isolated as described above. They were treated in AMD3100 (50 ng/mL), labeled with Di I (1,1′-dioctadecyl-3,3,3′,3′-tetramethylindocarbocyanine perchlorate, Molecular Probes) for 1 hour, and then injected into the right femoral vein of DB mice on post-wounding day 3. Intravascular PC dosage was based on prior calculation of circulating PCs following AMD3100 mobilization. Intravascular administration of DiI-labeled, Lin+ cells in wounded DB mice served as a control. Daily topical PDGF-BB treatment proceeded from time of injection until wound harvest on post-wounding days 7 and 21. Immediately prior to wound harvest, 100 μL of FITC-Tomato Lectin (Vector Labs) followed by 4% paraformaldehyde (Sigma) was injected into the right femoral vein of the mice. The wounds were then harvested for frozen sectioning as described above. Sections were DAPI stained and analyzed by fluorescent microscopy.

### Statistical Analysis

Data are presented as mean ± standard error of the mean. A one-way ANOVA with post-hoc Tukey Kramer was used for comparison of wound closure rates, cPC number, and vascular staining between all groups studied. A Student's t test was used for comparison between groups for the functional assays. Non-linear regression models and area-under-curve (AUC) analysis were performed using GraphPad Prism 5.0 software (San Diego, CA). Statistical significance was considered to be p<0.05. The number of mice per treatment group was determined using G*Power (Melbourne, Australia) to provide a power greater than 0.80. To determine if combination therapy (AMD3100 and PDGF-BB) had an additive or synergistic effect on wound healing, we used the Bliss method of analysis. Using the Bliss analysis, an additive effect on healing in response to the administration of two drugs that act independently is represented by Fa + Fb(1-Fa), where Fa represents the fractional response to drug “a” and Fb represents the fractional response to drug “b”. In our study, the Bliss formula may be re-written as the additive effect on healing  =  AMD3100 effect + (PDGF-BB effect)(1-AMD3100). If the observed healing is greater than the additive effect on healing, the combination therapy is considered to be synergistic.

## Results

### Impaired diabetic PC mobilization is rescued by AMD3100 treatment, but not PDGF-BB

**Figure 1:**
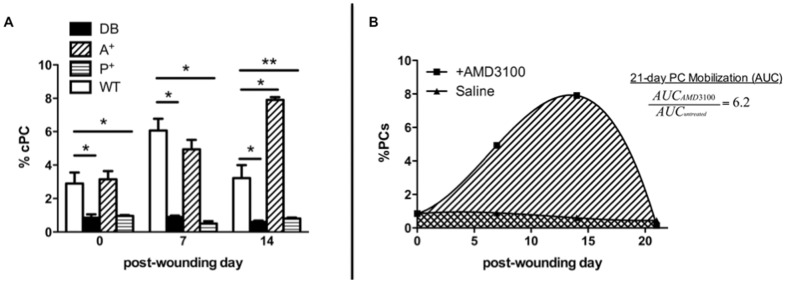
AMD3100 treatment (10 mg/kg IP), but not PDGF-BB (2 μg/wound topically), rescues the BM PC mobilization defect in wounded diabetic mice. Circulating(c) PC (L^-^S^+^K^+^) from wounded AMD3100-treated (A+), PDGF-BB treated (P+), or saline-treated (DB) db/db or wild-type (WT) mice were FACS-sorted from the circulating blood volume and quantified. A) Systemic AMD3100 mobilizes diabetic PCs at or above wild-type levels within the first two weeks post-injury while PDGF-BB does not alter diabetic PC mobilization. B) Over 3 weeks, area-under-curve analysis demonstrates a 6.2-fold increase in AMD3100-mediated BM PC-mobilization compared to saline-treated controls. (*p<0.05, **p<0.01 compared to wild-type control, values represent mean +/− SEM, 8 animals/group.)

In initial experiments, treatment of type II diabetic Leprdb/db (DB) mouse cutaneous wounds with PDGF-BB resulted in only a modest impact on wound closure rate. Surprised by this finding, we investigated the effects of PDGF-BB on BM PC mobilization. Using an established preclinical model of diabetic wound closure, we wounded DB and wild-type (WT) mice and treated them with saline or PDGF-BB. FACS analysis of circulating mononuclear cells of the wounded mice demonstrated that wounded DB mice consistently mobilize fewer PCs (L-S+K+ cells) at baseline, 7-, and 14-days post-wounding when compared to similarly wounded WT mice (0.85±0.3% vs. 2.9±0.7%, p<0.05; 0.9±0.1% vs. 6.1±0.7%, p = 0.02; and 0.6±0.1% vs. 3.2±0.8%, p = 0.03, respectively) (**FIGURE 1A**). Topical application of PDGF-BB had no appreciable effect on DB PC mobilization (0.97±0.3% vs. 0.85±0.3% at day 0, 0.4±0.1% vs. 0.9±0.1% at day 7, and 0.8±0.3% vs. 0.6±0.1% at day 14; p>0.05 for all).

Since topical PDGF-BB did not increase BM PC mobilization in DB mice, we examined the effects of intraperitoneal (i.p.) injection of AMD3100 in the cutaneous wound model. AMD3100 potentiated PC mobilization over 14-days when compared to saline-treated controls (3.1±0.9% vs. 0.85±0.3% at 1 hour, p<0.05; 4.9±1.0% vs. 0.9±0.1% at day 7, p<0.02; and 7.9±0.3% vs. 0.6±0.1% at day 14, p<0.02) (**FIGURE 1A**). Over 21 days, wounded DB mice treated with AMD3100 mobilized 6.2-fold more PCs than saline-treated DB controls (p<0.05) (**FIGURE 1B**).

### Only combination therapy normalizes wound neovascularization

**Figure 2:**
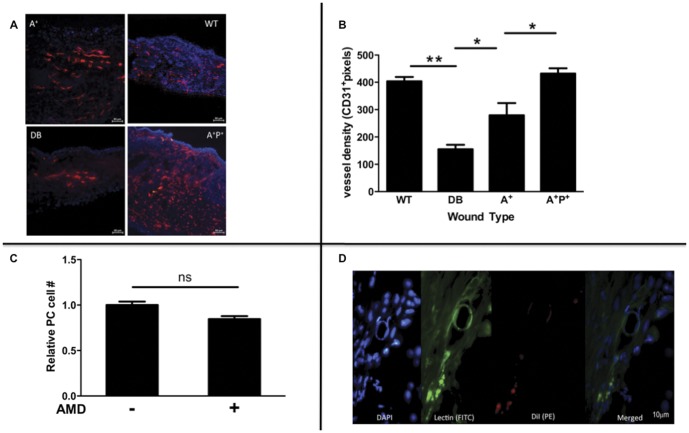
Combination therapy restores wound neovascularization partially though PC-mediated vasculogenesis. Wounds from AMD3100-treated (A+), AMD3100/PDGF-treated (A+P+), saline-treated diabetic (DB), and wild-type (WT) mice were harvested at post wounding day 21 for analysis. **A,B**) Immunofluorescent-staining of 28-day-old wounds for endothelial marker, CD31, demonstrates that only A+P+ therapy normalizes wound neovascularization to wild-type levels. **C**) BrdU-labeling of PCs in the presence of AMD3100 demonstrates only minor, non-significant, inhibition in PC proliferation (15.4±3.3% decrease, p = 0.06). **D**) When 2.5×10^4^ DiI-labeled PCs were intravascularly delivered to wounded animals on post-wounding day 1 and wounds were then harvested on post-wounding day 28 after lectin perfusion, we observe direct incorporation of PCs (red) into wound neovasculature (green) with DAPI counterstain (blue). (*p<0.05, **p<0.01 compared to wild-type control, values represent mean +/− SEM, 8 animals/group.)

Since DB mice have impaired wound closure and fewer cPCs after wounding, we examined the link between these two findings by determining the number of newly formed blood vessels in the wound (**FIGURE 2A**). CD31 immunofluorescence of DB wound tissue on day 28 demonstrated an average vessel density of 155.3±16 MESF/LPF. In contrast, at day 28, WT mice had 403.5±15.8 MESF/LPF. In spite of enhanced mobilization, treatment with AMD3100 increased wound neovascularization compared to non-treated DB mice to only 70% of WT levels (279.3±44.7 MESF/LPF v. 403.5±15.8 MESF/LPF; p<0.05) (**FIGURE 2B**). Day 28 wounds of PDGF-BB treated mice averaged 213.5±15.0 MESF/LPF, which was significantly more than DB wounds (p = 0.02) but not significantly different than AMD3100 treated mouse wounds. Interestingly, only combination therapy (AMD3100 + PDGF-BB) normalized wound neovascularization 431.8±19.3 pixels MESF/LPF. BrdU-labeling of PCs demonstrated that AMD3100 cytotoxicity did not significantly contribute to the less-than-expected levels of neovascularization (15.4±3.3% decrease, p = 0.06) (**FIGURE 2C**).

As it remains controversial whether cPCs directly participate in postnatal vasculogenesis, we labeled AMD3100 treated BM-derived PCs with a tracer, DiI, and injected them into the circulation of wounded diabetic mice treated with topical PDGF-BB. On post-wounding day 28, we observed direct incorporation of labeled PCs into the wound neovasculature, reinforcing the idea that BM PCs contribute to wound neovascularization (**FIGURE 2D**). By demonstrating that systemically administered BM-derived PCs incorporate into new vessels in the wound, we infer that AMD3100-mobilized endogenous PCs contribute to peripheral neovascularization in a similar mechanism.

### AMD3100-impaired PC migration can be overcome with PDGF-BB supplementation

**Figure 3:**
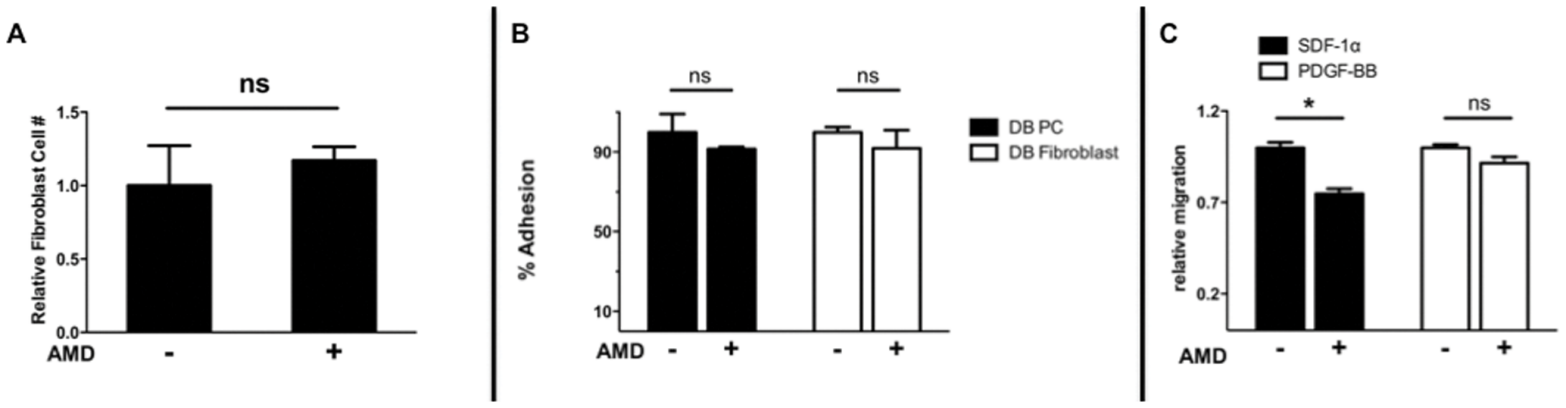
AMD3100 is PC-specific, altering PC migration towards SDF1α, but not towards PDGF-BB. 0.5×10^4^ PCs or primary db/db fibroblasts were plated in a 96-well plate, cultured in AMD3100-supplemented or control media for 3 days. **A**) BrdU staining of these cells reveals that AMD3100 does not significantly alter their proliferative capacity (p>0.05). **B**) Additionally, AMD3100 treatment of either cell line did not alter adhesion to fibronectin-coated chamber slides. **C**) After 5×10^4^ diabetic PCs were seeded on a fibronectin-coated 24-transwell insert with a receiver compartment containing media supplemented with either SDF1α (100 mg/ml) or PDGF-BB (100 ng/ml) and allowed to migrate for 20 hours, we found that AMD3100 treatment significantly impaired PC migration towards SDF1α (25% decrease, p<0.05) but not towards PDGF-BB (8.4% decrease, p>0.05). (*p<0.05, **p<0.01 compared to wild-type control, values represent mean +/− SEM, 8 animals/group.)

Surprised that AMD3100 treatment alone did not normalize wound neovascularization, we hypothesized that AMD3100 treatment competitively inhibited SDF1α-CXCR4 interactions vital to cPC trafficking to the wound. To explore this hypothesis, we used a modified Boyden-chamber assay to analyze PC migration (**FIGURE 3**). AMD3100 treatment inhibited PC migration towards SDF-1α (25.1±2.8% decline from control p<0.05). However, when PDGF-BB was supplemented to the receiver compartment, PC migration was restored despite the presence of AMD3100 (8.4%+4% decline, p>0.05).

### Combination therapy accelerates diabetic wound closure

**Figure 4:**
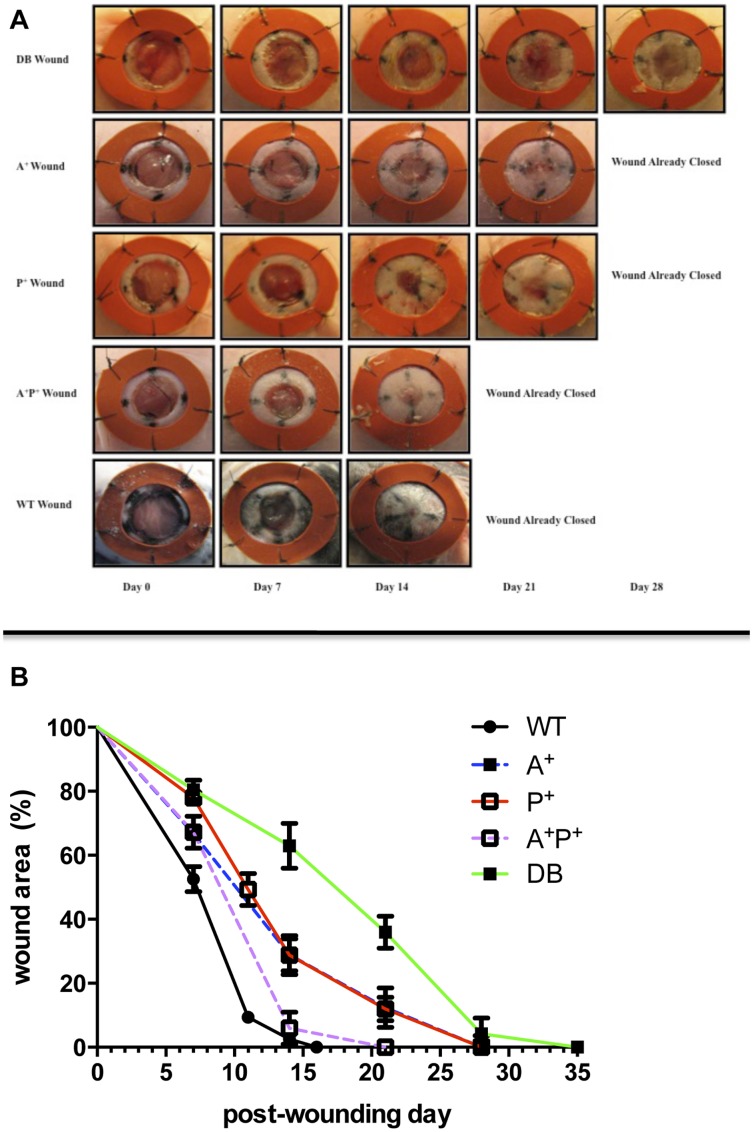
Combination therapy restores diabetic wound closure. Stented dorsal full-thickness dermal wounds were created on wild-type (WT) mice and diabetic mice treated with either saline (DB), AMD3100 (A+), PDGF-BB (P+), or a combination of both AMD3100 and PDGF-BB daily (A+P+). **A**) Representative photographs from each treatment group at days 0, 7, 14, 21, and 28 post-wounding. **B**) Using photogrammetric analysis, the percent wound closure was measured and compared between groups, with A+P+ mice showing wound healing rates comparable to WT mice. (*p<0.05, **p<0.01 compared to wild-type control, values represent mean +/− SEM, 8 animals/group.)

Using the stented-wound model, we observed that DB mice wound closure was significantly delayed in comparison to WT mice (19.6±2.0% vs. 47.4±4.4% closure at day 7, p<0.01; 37.1±9.0% vs. 98.4±0.8% closure at day 14, p<0.01; 64.0±9.0% vs. 100±0.0% closure at day 21, p = 0.02). While initially accelerating diabetic wound closure, single-agent treated mice (AMD3100 or PDGF), were statistically indistinguishable from saline-treated diabetic controls by day 28 (p = 0.51 and p = 0.49, respectively). In contrast, combination therapy (AMD3100/PDGF) reduced the diabetic wound closure time by an average of 11.4±2.3 days, approaching the WT phenotype (18.0±0.7 days vs. 15.0±1 days, respectively p<0.001)(**FIGURE 4**). Furthermore, functional assays with murine fibroblasts demonstrated that AMD3100 had no significant effects on their proliferation (**FIGURE 3A**) or adhesion (**FIGURE 3B**), suggesting that the observed improvement in regeneration was largely attributed to enhanced neovascularization.

## Discussion

There is significant evidence linking impaired neovascularization with delayed diabetic wound closure [13], [22], [23]. Consistent with our findings, several studies have implicated decreased cPCs as a causative factor [11], [13], [23]–[25]. Our prior work has demonstrated that an impaired SDF-1α switch mechanism within the marrow compartment impedes diabetic PC mobilization [11]. To illustrate this point, we previously transferred BM PCs directly into peripheral wounds, bypassing the mobilization defect, and enhancing diabetic wound closure [14]. However, as significant technical and practical barriers exist to the isolation, *ex vivo* expansion, and delivery of PCs, the future of this approach is limited [26], [27] Other recent studies have also overcome similar defects with both systemic and topical therapies [28], [29] While effective, these strategies are neither easily nor rapidly translatable to human subjects, as they are not approved for human use. Currently, we have proposed an endogenous strategy using FDA-approved drugs to improve diabetic wound closure.

AMD3100 was FDA-approved in 2008 for the mobilization of hematopoetic stem cells [27], [30]–[32]. Specifically, AMD3100 mobilizes hematopoetic stem cells by competitively binding to the cell surface CXCR4 receptor. Based on this mechanism of action, we hypothesized that AMD3100 treatment could accelerate diabetic wound closure by rescuing defective PC mobilization to augment wound neovascularization.

We first demonstrated that daily AMD3100 treatment increased diabetic PC mobilization over 6-fold, consistent with previous studies [11], [33], [34]. However, despite a 30% increase in PC mobilization over WT, AMD3100 treatment only partially-rescued diabetic wound neovascularization (70% of WT levels) – suggesting the possibility that AMD3100 treatment itself may impair PC trafficking/function, or that enhanced PC mobilization alone may not be sufficient to overcome the defects in diabetic neovascularization [35].

Based on these findings, and recognizing that AMD3100 may contribute to cellular dysfunction through CXCR4-SDF1α antagonism [36], we investigated the functional effects (proliferation, cellular-adhesion, and migratory capacity) of AMD3100 on diabetic fibroblasts and PCs. While we found no functional differences between AMD3100-treated diabetic fibroblasts and controls, we observed that AMD3100-treated PCs have impaired migration towards SDF1α [36] Hypothesizing that an AMD3100-induced impairment in the migratory ability of cPCs to home to the wound bed was the cause for the continued delay in closure seen in AMD3100-treated DB mice, we next investigated the addition of topical PDGF-BB to our treatment regimen.

Platelet-derived growth factor (PDGF-BB) is a 30 Kd protein involved in varied physiological processes including cell growth, progenitor cell migration and angiogenesis [37]. FDA approved in 1997, PDGF-BB remains the only growth factor available for the treatment of diabetic ulcers [38]. Previous reports on the efficacy of topical PDGF-BB on diabetic wound healing, however, have been equivocal [39], [40]. In our stented wound model, topical PDGF-BB modestly improved wound closure, however, without increasing PC mobilization. Interestingly, while PDGF-BB failed to in its ability to mobilize PCs, we observed that AMD3100-treated PCs maintained their migratory capacity to PDGF-BB. Given this finding, we hypothesized that a combination therapy to mobilize BM PCs (with AMD3100) and improve PC homing to the wound bed (with PDGF) would restore diabetic wound healing to wild-type rates.

In a similar study, Nishimura and colleagues detailed improvements in murine diabetic wound healing following a one-time, topical dose of AMD3100 (6 mg/kg) in a non-stented, excisional wound model [34]. While they did not report the time necessary for complete wound closure, they showed 2.5-fold acceleration in wound closure by 14-days, increased neovascularization, and increased cPCs 7-days post-wounding — consistent with our findings. Additionally, they found topical AMD3100 treatment to increase collagen-fiber formation, expression of SDF1α and PDGF-BB in the wound bed and fibroblast migration and proliferation. In contrast to the study by Nishimura et al., we systemically administered AMD3100 (10 mg/kg) on a daily basis in a stented excisional wound model and used smaller doses of AMD3100 in our in vitro studies (5–50 ng/ml vs. 2 μg/ml). This may account for the need to topically apply PDGF-BB in addition to the systemic administration of AMD3100 to completely correct the impaired wound healing in diabetic mice using our model. Topical application of AMD3100 undoubtedly results in higher concentrations for longer periods of time in the wound bed compared to its systemic administration. Perhaps, this is why our study cannot corroborate their findings of AMD3100 induced increases in fibroblast proliferation. Taken together, we believe our findings further refine the model of Nishimura et al. and suggest that AMD3100 mobilizes CXCR4+ PCs for which PDGF-BB is a dominant chemotactic signal contributing to PC homing to and engraftment in the wound.

Addressing defects in both PC mobilization and trafficking, we observed that AMD3100/PDGF-BB combination therapy synergistically rescued diabetic wound closure, approaching the wild-type healing trajectory. As a single-agent, both AMD3100 and PDGF-BB accelerated wound closure by approximately 20% individually; used in combination, their effects were synergistic (calculated by the Bliss method) resulting in approximately 40% reduction in time required for wound closure. A recent publication by Sciaccaluga et al. may provide insight into this finding [41]. Specifically, they observed that the interaction between PDGFR and CXCR4 is essential in glioblastoma cell chemotaxis [41]. Although not evaluated in our study, cPC migration may be augmented via a similar mechanism. Further studies are needed to determine whether the synergistic effect in wound healing seen in our model with the topical application of PDGF-BB and systemic administration of AMD3100 is a result of crosstalk between the PDGF-BB/PDGFR and CXCR4/CXCL12 pathways.

Histologically, combination therapy was characterized by supra-normal neovascularization at 28-days post-wounding. It is noteworthy to report that only daily AMD3100/PDGF-BB treatment regimens improved wound closure; a one-time dose of AMD3100/PDGF-BB failed to substantially augment wound closure rates (data not shown). Why daily mobilization of BM PCs is necessary to improve diabetic wound closure remains an important question that requires further study. Previously we have shown that repeated delivery of VEGF was necessary to improve diabetic wound closure [22]. As diabetic-related cellular defects may retard regeneration, extended periods of PC mobilization may be required for adequate tissue repair and neovascularization. Additionally, circulating PCs are known to rapidly return to the BM and/or extramedullary sites following mobilization [42] and thus it may not be surprising that we did not observe effects on wound closure after a single treatment.

There are several limitations to our study. Specifically, we do not directly show an increased number of PCs in the wounds of diabetic mice treated with the combination of AMD3100 and PDGF-BB, and we do not rule out other mechanisms by which this therapy may improve wound healing independent of cPCs recruitment. In fact, in a choroidal neovascularization model, CXCR4 inhibition with AMD3100 was found to be anti-angiogenic when given continuously (30 mg/kg/d) starting at the time of vascular insult [43] In the same model, however, AMD3100 was ineffective as an anti-angiogenic factor if treatment was delayed two weeks following the vascular insult. Although there is some consensus that PCs aid in wound closure [44], [45], it is controversial as to whether PCs directly participate in postnatal vasculogenesis or simply coordinate neovascularization through indirect/paracrine interactions [8], [10]. CXCR4 signaling, in particular, has been shown to induce the upregulation of VEGF and other chemokines that contribute to angiogenesis via alternate pathways (non-CXCR4/SDF-1 mediated) [46]. While we did not directly assess indirect mechanisms of PC-mediated neovascularization in our study, we demonstrate that PCs migrate to diabetic wounds, engraft, and directly participate in vasculogenesis. This was confirmed by immunohistology of wounds after systemic injection of DiI-labeled PCs into DB mice following wounding. Future studies investigating PC engraftment as well as the levels of angiogenic chemokines (e.g. VEGF) in these diabteric wounds following treatment with AMD3100/PDGF-BB will help elucidate the exact mechanisms involved in the improved wound healing seen in this study.

Combination AMD3100/PDGF-BB treatment rescues diabetic wound closure by improving PC mobilization and trafficking to cutaneous wounds. Specifically, we show that AMD3100 therapy rescues a diabetes-specific defect in PC mobilization; however, the addition topical PDGF-BB is required to normalize PC homing/engraftment into the wound. The marked efficacy of this therapeutic strategy in a preclinical model of diabetic wound healing, lends itself to rapid translation to human trials.
